# Standard screening methods underreport AAV-mediated transduction and gene editing

**DOI:** 10.1038/s41467-019-11321-7

**Published:** 2019-07-30

**Authors:** Jonathan F. Lang, Sushila A. Toulmin, Kasey L. Brida, Laurence C. Eisenlohr, Beverly L. Davidson

**Affiliations:** 1 The Children’s Hospital of Philadelphia, Philadelphia, PA 19104 USA; 2 The Department of Pathology and Laboratory Medicine, Perelman School of Medicine, University of Pennsylvania, Philadelphia, PA 19104 USA

**Keywords:** CRISPR-Cas9 genome editing, Molecular medicine

## Abstract

Conventional methods to discern adeno-associated virus (AAV) vector transduction patterns are based on high, stable expression of a reporter gene. As a consequence, conventionally described tropisms omit cell types that undergo transient transduction, or have low but undetectable levels of reporter expression. This creates a blind spot for AAV-based genome editing applications because only minimal transgene expression is required for activity. Here, we use editing-reporter mice to fill this void. Our approach sensitively captures both high and low transgene expression from AAV vectors. Using AAV8 and other serotypes, we demonstrate the superiority of the approach in a side-by-side comparison with traditional methods, demonstrate numerous, previously unknown sites of AAV targeting, and better predict the gene editing footprint after AAV-CRISPR delivery. We anticipate that this system, which captures the full spectrum of transduction patterns from AAV vectors in vivo, will be foundational to current and emerging AAV technologies.

## Abstract (web summary)

Conventional methods to detect AAV vector transduction can miss transient or low levels of reporter expression. Here the authors use editing-reporter mice and discover numerous sites of AAV targeting along with better prediction of the gene editing footprint.

## Introduction

Safe and effective applications of adeno-associated viral (AAV) vectors for human disease therapies require that we understand their biodistribution, and additionally, their ability to drive transgene expression. AAV tropisms are typically defined by evaluating tissues several weeks after reporter-encoding (e.g. eGFP or lacZ) recombinant AAVs are delivered intravascularly (IV) into the cerebral spinal fluid (CSF) or directly to organs of interest. Given the same expression cassette, different AAV capsids produce varying patterns of transgene expression in host tissues, and these reporter-based transduction profiles have been well-described^1–4^. Classic methods work well for applications that desire sustained, high transgene expression. For example, systemic AAV8 delivery shows strong liver expression promoting its use for liver-targeted therapies^5^.

The discovery of the CRISPR-Cas9 system has revolutionized genome editing in biomedical research, but in vivo applications are limited by currently available modes of delivery. Because AAV has a well-defined in vivo safety profile, there is substantial interest in using AAV to deliver CRISPR-Cas9 genome editing machinery^6–9^. Conventionally described AAV tropisms have been used to guide these applications, but these data fail to report low level, or, relevant to editing, transient transgene expression. Minimal levels of CRISPR-Cas9 machinery can induce permanent genome modification, and so, even low expression from AAV vectors could provide a risk if expressed in unintended tissues.

Here, we harness editing-dependent reporter mice and show that AAV8 and other serotypes readily transduce cells in the kidney and spleen, in addition to the liver. Moreover, at relatively low vector doses nearly all hepatocytes show evidence of low level, transient expression sufficient for genome editing. The enhanced sensitivity of this approach also reveals previously undescribed sites of AAV transduction for vectors harboring presumed cell and tissue specific promoters. Cumulatively the data reveal expanded and unexpected tropisms following AAV delivery that will help guide gene therapy studies and translational applications.

## Results

### Optimized screen for assessing AAV liver transduction

**Fig. 1:**
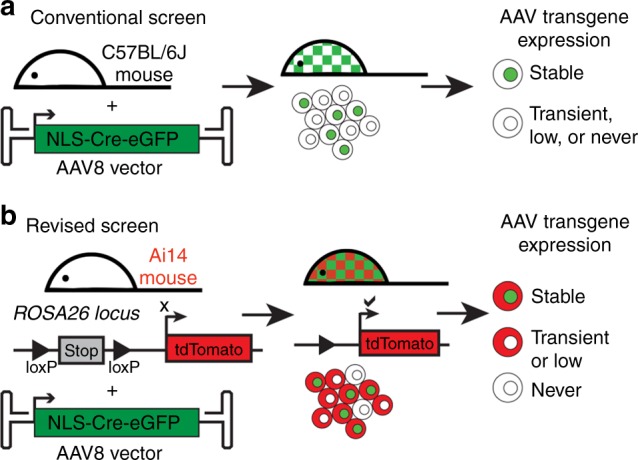
Comparison of conventional and revised AAV tropism screening methods. **a** Following injection of AAV2/8.CMV.HI.eGFP-Cre.WPRE.SV40 (AAV8-Cre-eGFP) into C57BL/6J mice, only the subset of cells with stable, high transgene expression will show nuclear-localized eGFP. Cells with transient or low transgene expression are missed. **b** Injection of the same AAV8-Cre-eGFP vector into Ai14 reporter mice (containing an endogenous loxP-STOP-loxP-tdTomato locus) captures cells with stable, high transgene expression (green nuclei) as well as cells with transient or low expression of the AAV transgenes (Cre-tagged tdTomato positive cells)

Conventional screens capture transduced cells with stable, high expression of an encoded reporter transgene. To replicate this established strategy, we performed systemic injections of AAV2/8.CMV.HI.eGFP-Cre.WPRE.SV40 (AAV8-Cre-eGFP) vector into C57BL/6J mice. Cells with stable, high expression of the AAV transgenes show nuclear-localized eGFP (Fig. 1a).

We hypothesized that injection of the same vector into a loxP-STOP-loxP-tdTomato (Ai14) transgenic reporter mouse would capture both stable, high transgene expression as well as transient or low transgene expression^10^. In Ai14 mice, robust, stable transgene expression generates nuclear-localized eGFP and Cre mediates recombination and activation of the endogenous tdTomato reporter. Transient or low levels of Cre:eGFP, which may be insufficient for detectable nuclear-localized eGFP, can still activate endogenous tdTomato (Fig. 1b).

To test this hypothesis, three doses of AAV8-Cre-eGFP (1e10, 1e11, and 3.16e11 total vector genomes (vg)) were infused into adult, male Ai14 and C57BL/6J mice. Ai14 and C57BL/6J mice share the same genetic background, and thus would not differ in AAV8 attachment or cell entry. Mice were euthanized 2 weeks later and tissues were collected for histology.

**Fig. 2:**
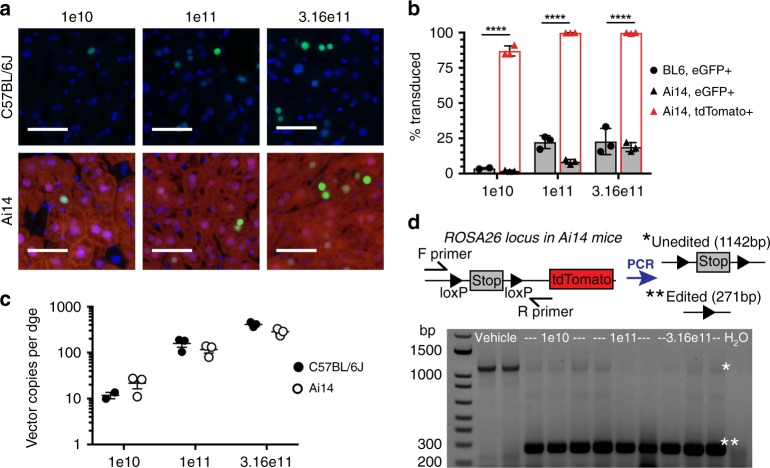
Ai14 mice improve the sensitivity for liver AAV transduction. **a** Representative photomicrographs of liver sections harvested from AAV8-Cre-eGFP treated C57BL/6J or Ai14 mice 2 weeks post IV delivery at the indicated doses (total vector genomes per mouse). eGFP (green) and tdTomato expression (red) and nuclei (blue) are evident. *n* = 2–3 for C57BL/6J mice, *n* = 3 for Ai14 mice. Scale bars = 50 µm. **b** Quantification of hepatocyte transduction in C57BL/6J and Ai14 at the indicated doses. Values for individual mice are shown along with the mean and standard deviation for each strain and dose group. Data were analyzed by two way-ANOVA followed by Bonferroni post hoc comparing % transduced cells per animal in BL6 (%eGFP+) versus Ai14 (%tdTomato+) mice, ****p<0.0001. **c** Comparison of AAV vector copies per diploid genome equivalent (dge) in liver samples. Values for individual mice are shown along with the mean and standard error for each strain and dose group. **d** Cre-mediated recombination in Ai14 mice at the *ROSA26* locus as detected by PCR assay of liver genomic DNA. Source data are provided as a Source Data file

AAV8 has been used extensively in preclinical and human clinical trials for liver-targeted gene therapy^5,11–13^. After intravenous injection, AAV8 accumulates at high copy number in the liver in a dose-responsive manner^12^. As expected, both C57BL/6J and Ai14 mice demonstrated dose-responsive nuclear eGFP expression (Fig. 2a). The Ai14 mice, however, also captured dose-responsive tdTomato expression due to the AAV-delivered Cre transgene. The percent transduced cells were measured in both settings and compared (% eGFP+ in the C57BL/6J mice versus % tdTomato+ in the Ai14 mice) (Fig. 2b). Ai14 mice revealed significantly greater levels of transduction than was noted in C57BL/6J mice. The large discrepancy between eGFP+ versus tdTomato+ cells in Ai14 mice suggests that nearly all hepatocytes at least transiently express Cre:eGFP, even at low doses, but only a subset achieves stable, high transgene expression.

To compare uptake of AAV8 vector in the two mouse strains, total DNA was isolated from liver tissues and AAV vector copies per diploid genome equivalent (dge) was measured. The two strains had similar vector copies per dge at each dose, suggesting that vector uptake is not responsible for the discrepancy between the two mouse strains in measurable transient or low level AAV transduction (Fig. 2c).

Recombination at the *ROSA26* locus (containing the loxP-STOP-loxP-tdTomato reporter) captures Cre activity independent of fluorescent protein levels. As expected, genomic DNA from vehicle-injected Ai14 mouse livers showed only the unedited ROSA26 band while genomic DNA from AAV8-Cre-eGFP-injected Ai14 mouse livers showed primarily the edited *ROSA26* band as assessed by DNA PCR (Fig. 2d). These results are consistent with the abundant tdTomato expression in the livers of the Ai14 mice.

### AAV8 transduces glomerular cells in mouse kidney

**Fig. 3:**
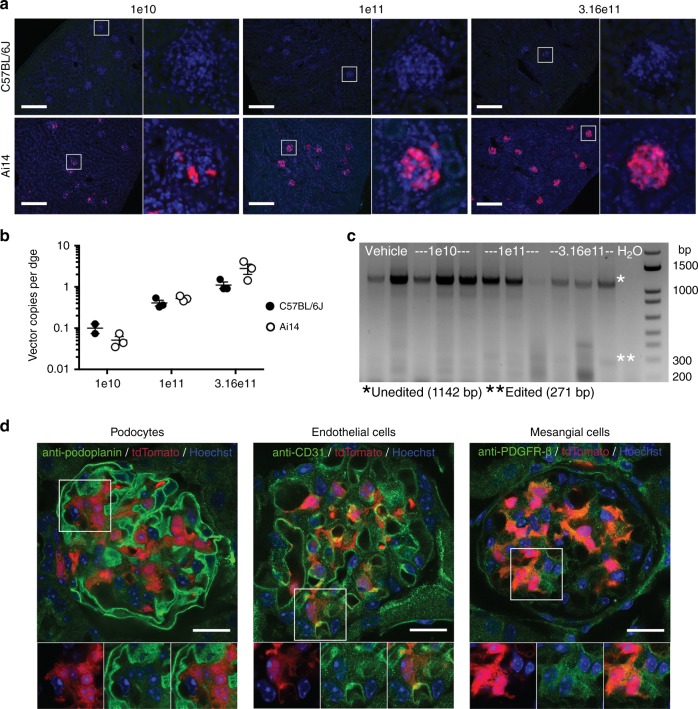
AAV8 transduces kidney glomeruli. **a** Representative fluorescent photomicrographs of kidney sections from C57BL/6J and Ai14 mice 2 weeks after IV delivery of AAV8-Cre-eGFP at the indicated doses. No eGFP expression is detected in green channel in either mouse strain for all doses tested. tdTomato (red) indicates editing; nuclei are shown (blue). *n* = 2–3 for C57BL/6J mice, *n* = 3 for Ai14 mice. Scale bars = 250 µm. **b** Comparison of AAV vector copies per diploid genome equivalent (dge) in kidney samples from C57BL/6J and Ai14 mice 2 weeks post IV delivery. Values for individual mice are shown along with the mean and standard error for each strain and dose group. **c** Cre-mediated recombination at the *ROSA26* locus as detected by PCR assay of kidney genomic DNA from Ai14 mice. **d** Immunohistochemistry for glomerular cell types in Ai14 mice 2 weeks after AAV8-Cre-eGFP delivery (3.16e11 vg). Primary antibodies labeled podocytes (anti-podoplanin), endothelial cells (anti-CD31), and mesangial cells (anti-PDGFR-β) and were detected with Alexa-488 conjugated secondary antibodies (green). Cre-tagged cells are tdTomato positive. Scale bars = 25 µm. Source data are provided as a Source Data file

AAV8 vector DNA accumulates at low levels in mouse kidney following intravenous injection, but embedded transgene cassettes are not notably expressed^2,11,14^. The C57BL/6J mice injected with AAV8-Cre-eGFP recapitulated these findings, demonstrating no detectable eGFP expression in the kidney at these vector doses and time point (Fig. 3a), despite containing AAV vector DNA genomes (Fig. 3b) to levels comparable to those previously reported^11,12^. In contrast, AAV8-Cre-eGFP-injected Ai14 mice revealed strong, dose-dependent editing in kidney glomeruli (Fig. 3a). The difference in detectable Cre-eGFP transgene expression in the Ai14 but not C57BL/6J mice was not due to variation in vector copies between the two mouse strains; both showed similar vector copies per dge in kidney in the three dose groups (Fig. 3b). To assess editing, whole kidney was lysed and PCR done on genomic DNA. The expected edited *ROSA26* band was evident in samples from kidney genomic DNA harvested from mice treated at the medium and highest vector doses (Fig. 3c), but not at lower doses. This is likely to due to the fact that the edited glomerular cells were diluted by unedited cells in the kidney isolates.

The glomerulus is comprised of podocytes, endothelial cells, and mesangial cells and identifying which cell types are transduced by AAV8 may prove useful to future gene therapy studies. Immunofluorescence experiments confirmed that AAV8-transduced cells (tdTomato positive) colocalized with endothelial cell (CD31) and mesangial cell markers (PDGFR-ß), but not with a podocyte marker (podoplanin) (Fig. 3d). This suggests that AAV8 transduces both endothelial and mesangial cells in the mouse glomerulus.

### AAV8 transduces multiple cell types in mouse spleen

**Fig. 4:**
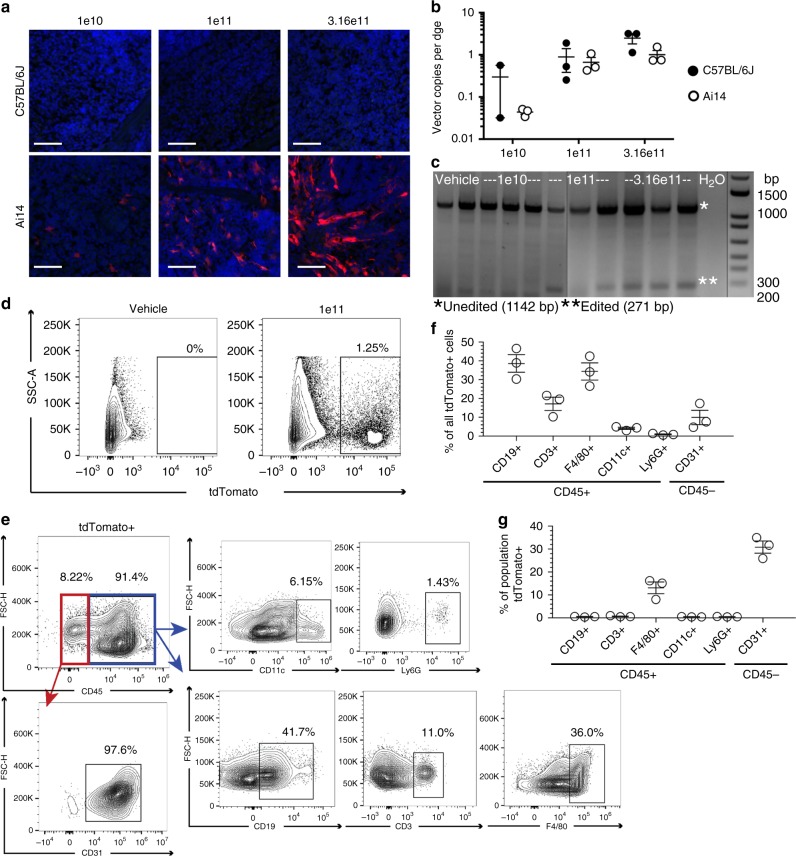
AAV8 tropism in mouse spleen. **a** Representative fluorescent photomicrographs of spleen sections from C57BL/6J and Ai14 mice 2 weeks after IV delivery of AAV8-Cre-eGFP at the indicated doses. No eGFP expression is detected in green channel in either mouse strain for all doses tested. tdTomato (red) indicates editing; nuclei are shown (blue). *n* = 2–3 for C57BL/6J mice, *n* = 3 for Ai14 mice. Scale bars = 50 µm. **b** Comparison of AAV vector copies per diploid genome equivalent (dge) in spleen samples from C57BL/6J and Ai14 mice 2 weeks post IV delivery. Values for individual mice are shown along with the mean and standard error for each strain and dose group. **c** Cre-mediated recombination at the *ROSA26* locus is detected via PCR assay of spleen genomic DNA from Ai14 mice. **d** Flow cytometry was used to measure editing (tdTomato+) in single, live splenocytes isolated from vehicle- or AAV-injected Ai14 mice. *n* = 5 for both groups. **e** Splenocytes were co-stained for relevant cell markers and quantified via flow. Representative plots are shown from the AAV-injected group. *n* = 3 for each antibody co-stain. Gates for each marker were established with fluorescence minus one controls. **f** Plot showing the contributions of different cell types to the total tdTomato+ population. *n* = 3 mice for each cell type with mean and standard error shown. **g** Plot showing the percent of each cell type that was tdTomato+. *n* = 3 mice for each cell type with mean and standard error shown. Specific marker fluorochromes are indicated in Methods. Source data are provided as a Source Data file

Consistent with other published work, AAV8-Cre-eGFP does not appear to transduce C57BL/6J mouse spleen as no notable eGFP expression was detected at any dose (Fig. 4a)^12,14,15^. In Ai14 mice, however, AAV8-Cre-eGFP displays clear dose-responsive transduction in the spleen (Fig. 4a). The detection of AAV8 transgene expression in the spleens of Ai14 but not C57BL/6J mice was likely not due to differences in AAV8 vector copies; AAV8 vector copies per dge in the C57BL/6J mice was comparable to the Ai14 mice and still did not produce detectable eGFP expression (Fig. 4b). Again, PCR assay of the *ROSA26* locus from spleen genomic DNA showed clear editing at the medium and highest doses of vector in the Ai14 mice (Fig. 4c).

A higher resolution picture of the AAV8-mediated mouse spleen transduction in Ai14 mice was captured using flow cytometry. As expected, a clear transduced (tdTomato positive) cell population was present in the AAV8-injected but not vehicle-injected animals (Fig. 4d). The spleen comprises hematopoietic (CD45+) and non-hematopoietic (CD45-) cells, with CD45+ cells including B cells (CD19+), T cells (CD3+), macrophages (F4/80+), dendritic cells (CD11c+), and granulocytes (Ly6G+) among others. Non-hematopoietic cells in the spleen include endothelial cells (CD31+) and other cell types^16^. Analysis of a representative AAV8-injected Ai14 mouse spleen revealed that the tdTomato-positive cell population was 91.4% CD45+ and 8.22% CD45− (Fig. 4e). The tdTomato+/CD45+ population was co-positive for a variety of other cell markers including CD19, CD3, F4/80, CD11c, and Ly6G, indicating that AAV8-transduced B cells, T cells, macrophages, dendritic cells, and granulocytes (Fig. 4e). AAV8 also transduced splenic endothelial cells as the tdTomato+/CD45− population was almost entirely CD31+ (Fig. 4e). An example of the full gating strategy is available (Supplementary Fig. 1).

By raw numbers, the largest contributors to the tdTomato-positive cell population were CD45+/CD19+ (B cells) and CD45+/F4/80+ (macrophages) (Fig. 4f). We further calculated the percent of cell type (e.g. B cells) that was tdTomato+. Interestingly, by this criterion, macrophages and endothelial cells were more highly transduced as a population by AAV8 than any of the other spleen cell types analyzed (Fig. 4g).

### Expanded sites of transduction revealed in additional tissues

**Fig. 5:**
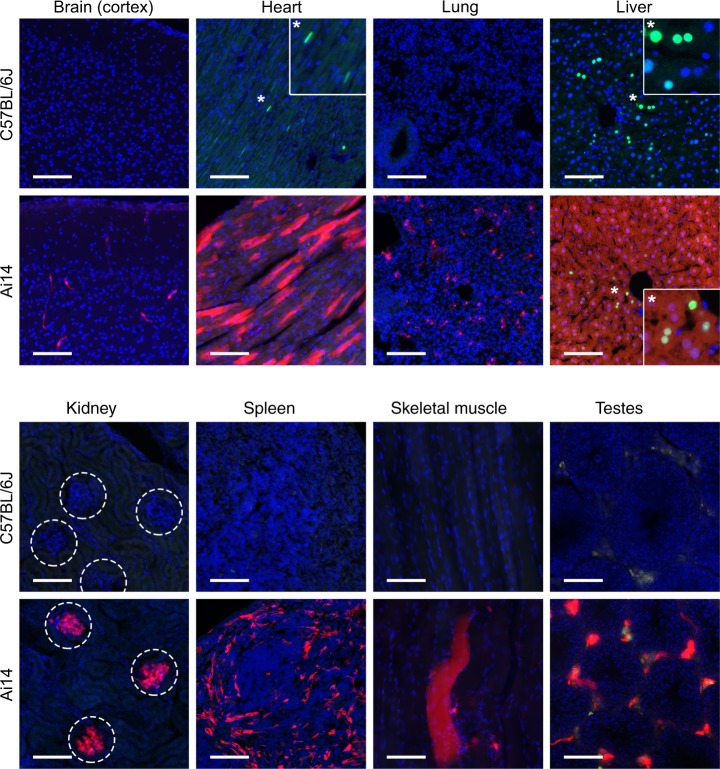
Ai14 mice capture AAV8 transgene expression across tissues. Representative photomicrographs of tissue from C57BL/6J or Ai14 mice at 2 weeks post IV delivery of 3.16e11 vg AAV8-Cre-eGFP per mouse. eGFP (green), tdTomato expression (red), and nuclei (blue) are shown. *n* = 3 for C57BL/6J and Ai14 mice. Insets in heart and liver highlight nuclear eGFP expression. Dashed circles highlight glomeruli. Autofluorescent material is present in testes. Scale bars = 100 µm

Having already examined liver, kidney, and spleen, other tissues commonly surveyed in AAV tropism studies were analyzed. As expected, C57BL/6J mice showed nuclear eGFP expression in tissues of known AAV8 tropism (heart and liver) but did not show detectable eGFP expression in any of the other tissues surveyed (Fig. 5)^11,12^. The Ai14 mice captured transduction in heart and liver, but by contrast also showed tdTomato expression in all tissues analyzed (Fig. 5). Vehicle-injected control Ai14 mice did not result in detectable tdTomato to any appreciable level (Supplementary Fig. 2).

Different combinations of AAV serotypes, transgenes, and transgenic editing-reporter mice were used to more thoroughly test the hypothesis that conventional screens underreport AAV-mediated transduction. Using the Ai14 reporter mice, we found that AAV5-Cre-eGFP and AAV9-Cre-eGFP (Supplementary Fig. 3) transduced these same tissues. Also, we inverted the reporters by delivering AAV8-Cre-mCherry into Ai6 (ZsGreen) mice (Supplementary Fig. 4a). The detected transgene expression from AAV8-Cre-mCherry (which contained a related promoter to AAV8-Cre-eGFP) in the Ai6 mice mirrored those in the Ai14 mice, supporting the robustness of the method (Supplementary Fig. 4b).

Next, we evaluated “tissue specific” promoters. For this, AAV8-LSP-Cre-mCherry (expression driven by a liver-specific promoter) was injected into Ai6 mice. While liver transgene expression was greater, there remained glomerular transduction albeit to reduced levels than noted earlier. Also of note, there remained considerable transgene expression in the spleen (Supplementary Fig. 4c).

### AAV8-CRISPR-Cas9 editing is captured across tissues

**Fig. 6:**
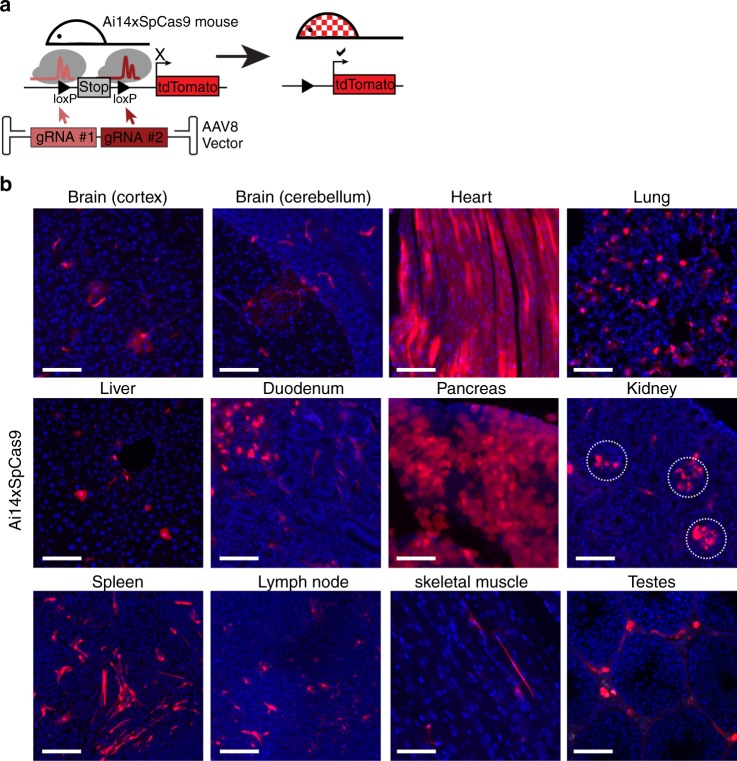
Ai14xSpcas9 mice capture AAV8-CRISPR-Cas9 editing across tissues. **a** AAV8-ROSA-gRNAs vector edits the *ROSA26* locus and activates tdTomato expression in Ai14xSpCas9 mice. **b** Representative photomicrographs of tissue from Ai14xSpCas9 mice at 2 weeks post IV delivery of 2.3e11 vg AAV8-ROSA-gRNAs per mouse. tdTomato expression (red) and nuclei (blue) are shown. *n* = 3 Ai14xSpCas9 mice. Scale bars = 100 µm

We tested the sensitivity of the system for detecting CRISPR/Cas editing using nuclease active SpCas9. A single vector encoding two guide RNAs (AAV8-ROSA26-gRNAs) targeting the stop cassette from the *ROSA26* locus (Fig. 6a) was infused into Ai14xSpCas9 mice at a dose of 2.3e11 vg per mouse. These mice contained the tdTomato reporter allele at the *ROSA26* locus and constitutive, whole body SpCas9 expression. Non-injected control Ai14xSpCas9 mice had rare to undetectable tdTomato-positive cells in liver sections (Supplementary Fig. 5). As expected, gene editing (tdTomato expression) was detected in the liver and heart of Ai14xSpCas9 mice at 2 weeks post AAV8-ROSA26-gRNAs injection (Fig. 6b). However, in a manner similar to AAV-Cre-eGFP, widespread CRISPR-Cas9 editing was detected across brain, heart, lung, liver, kidney, spleen, skeletal muscle, and testes (Fig. 6b). We also detected CRISPR-Cas9 editing in duodenum, pancreas, and lymph node in the Ai14xSpCas9 mice (Fig. 6b), sites that we did not examine with the Cre-eGFP vector.

## Discussion

Typically described AAV tropisms fail to capture cells with low or transient reporter gene expression. For genome editing machinery like Cre or CRISPR/Cas9, only a small amount of expression is required for a permanent alteration of host DNA. Clearly, methods that better record the full transcriptional history of AAV vector DNA are essential for genome editing applications of AAV. To address this problem, we present a method to capture low and/or transient AAV transgene expression. This method improves detection of AAV expression within tissues of known tropism, uncovers additional sites of AAV expression, and highlights other organs and cell types that may be subject to gene editing after AAV delivery.

In mouse liver, which is highly transduced by AAV8, the Ai14 and Ai6 mice significantly improve detection of AAV transgene expression (Fig. 2). Previous work (at similar vector doses) has shown that nearly all hepatocytes internalize systemically administered AAV vector within 24 h post-injection^17^. Until now, the transcriptional fate of that AAV vector DNA has not been thoroughly captured. We show that at commonly used AAV8 vector doses, nearly all hepatocytes express vector DNA at levels sufficient for gene editing. Interestingly, only a subset of hepatocytes that are tagged by transient transgene expression eventually demonstrate stable expression. Earlier work hinted at this possibility in the liver, but did not demonstrate the extent of transient expression across tissues^13^.

One concern of CRISPR-Cas9-based editing is the development of immune responses against Cas9 protein^18^, which could be avoided if expression was transient. Indeed, our data support that high, stable transgene expression is not necessary for effective editing (Fig. 2). Thus, using doses that are suboptimal for stable transgene expression may be optimal for genome editing strategies. Additionally, self-deleting AAV-CRISPR systems may permit transient exposure to the Cas9 proteins^19^.

AAV8 is deemed a highly efficient vector for mouse liver transduction, with no known tropism for spleen or kidney; however, we show robust tdTomato tagging of these two tissues. Circulating tdTomato released from transduced cells and taken up by spleen and kidney cells are not likely, as editing was obvious in these tissues at the DNA level. Additionally, AAV vector DNA was detected in a dose-dependent manner and the pattern of tdTomato fluorescence was limited to distinct cells at each dose. Also, the observed tropisms were maintained with an inverted reporter system and changed with an alternative promoter. Finally, a completely different editing strategy (CRISPR/Cas9) highlighted similar patterns of transduction.

Developing AAVs that target particular cell types or substructures of kidney and spleen would greatly expand the utility of the AAV platform. In kidney, there have been several efforts to improve AAV-mediated gene therapy with alternative routes of administration (retro-urethral, direct parenchymal injection) or the use of AAV9 (refs. ^20–23^). In all cases, AAV-mediated transgene expression was low and or non-specific. More recently, Cre-dependent reporter mice were used to demonstrate that AAV-Anc80 transduces glomerular cells including mesangial cells^24^. Our work shows that AAV8 also targets glomerular endothelial and mesangial cells without major transduction of other kidney cell types (Fig. 3).

Although AAV8 vector DNA accumulates after IV injection in the spleen, transgene expression has not been demonstrated^11,12,14^. Our approach amplifies low levels of AAV transgene expression and clearly captures spleen transduction with constitutive and “tissue specific” promoters. The possibility that “tissue specific” promoters can be leaky enough for gene editing in off-target tissues is a potential confounding factor for future gene editing approaches. Additionally, while the immunologic consequences of this transgene expression in spleen is unknown, it may catalyze future work to understand immune responses to AAV vectors.

One limitation in this screening method is that cell division of targeted progenitor/stem cells would cause an overestimation of AAV transduction. While we did not assess progenitor/stem cell transduction in this work, a recent study showed that AAV does have the potential to transduce hematopoietic, satellite, and mesenchymal progenitor cells^25^. The short, 2-week post-injection time period used here reduces the likelihood that this contributed significantly to our findings.

It is important to identify sites of transient/low AAV transgene expression when delivering gene editing machinery via AAV. Interestingly, in some tissues, CRISPR-Cas9 editing showed superior sensitivity to Cre-eGFP; we demonstrate, based on morphological criteria, neuron and astrocyte transduction across the cortex, a finding not observed with Cre-eGFP. In other tissues, Cre-eGFP was superior to CRISPR/Cas9, with nearly complete hepatocyte transduction at a dose of 1e10 vg AAV-Cre-eGFP per mouse while only scattered hepatocytes were tagged by editing using 2.3e11 vg of AAV-gRNA. This may reflect that repair outcomes vary by tissue after AAV-CRISPR/Cas9-mediated double-stranded break formation^9^. Nevertheless, both the AAV-Cre-eGFP and AAV-CRISPR-Cas9 systems used here define additional, previously unknown sites and cells that are transduced by AAV.

In sum, these results show that AAV-mediated transgene expression has been underestimated by historical methods. We provide an approach that better reveals the true extent of AAV-mediated gene transfer, which will be crucial for genome editing applications of AAV and future studies of AAV vector biology.

## Methods

### Animals

All animal procedures were approved by The Children’s Hospital of Philadelphia Institutional Animal Care and Use Committee. C57BL/6J (C57BL/6J, Stock No: #000664), Ai14 (B6.Cg-Gt(ROSA)26Sor^tm14(CAG-tdTomato)Hze^/J, Stock No: #007914)^10^, Ai6 (B6.Cg-Gt(ROSA)26Sor^tm6(CAG-ZsGreen1)Hze^/J, Stock No: #007906)^10^, and SpCas9 (B6J.129(Cg)-Igs2^tm1.1(CAG-cas9*)Mmw^/J, Stock No: #028239)^26^ mice were obtained from Jackson Laboratories. Ai14 and SpCas9 mice were crossed to generate Ai14xSpCas9 offspring (each containing one copy of a tdTomato reporter allele and one copy of a constitutive SpCas9 expression allele). Mice were housed in an enriched, temperature-controlled environment on a 12-h light/dark cycle. Food and water were provided ad libitum. All studies were performed with adult (>9 weeks old) male mice.

### AAV vectors

The AAV5-, AAV8-, and AAV9-Cre-eGFP vector used in this study were purchased from Addgene (#105545-AAV5, -AAV8, and -AAV9; http://n2t.net/addgene:105545; RRID:Addgene_105545). Gene expression is driven by a CMV enhancer/promoter fused to intron 1 of human growth hormone. Vector titer was determined by digital droplet PCR and recorded in genome copies (GC) per ml. GC per ml is equivalent to vg per ml.

For vector production, we used polyethylenimine Max (PEI Max, Polysciences) triple transfection of HEK293 cells with subsequent purification via iodixanol gradient ultracentrifugation^27^. After isolation from producer cells and centrifugation as described in detail in ^27^, samples were concentrated using a Vivaspin 6 concentrator (MWCO 100K; Vivaproducts VSO641, Littleton, MA 01460). All viruses were concentrated in vector stock buffer (180 mM NaCl, 10 mM Sodium phosphate dibasic, 0.001% Pluronic F68, pH 7.4). The AAV8-ROSA26-gRNA vector was cloned using previously described gRNA sequences^7^ (5′-AAAGAATTGATTTGATACCG-3′ and 5′–GTATGCTATACGAAGTTATT-3′) downstream separate U6 promoters. The AAV8-LSP-Cre-mCherry vector expresses Cre from a liver-specific promoter (LSP) that contains a single human ApoE enhancer region and a human alpha1-antitrypsin promoter. Vector titers were determined by qPCR and recorded in vg/ml.

AAV8-CMV-Cre-mCherry was purchased from the Viral Vector Core at the University of Iowa (http://www.medicine.uiowa.edu/vectorcore, AAV2/8CMVCre-mCherry, lot: AAV2919). Vector titer was determined by qPCR and recorded in vg/ml.

### Mouse procedures

For systemic AAV injections, mice were anesthetized with isoflurane and the right jugular vein was surgically exposed. AAV vector was diluted up to a volume of 100 µl using vector dilution buffer (0.01 M sodium phosphate dibasic, 0.18 M sodium chloride, and 0.001% Pluronic F-68 dialysis buffer, pH = 7.4) according to the desired dose (1e10, 1e11, or 3.16e11 total vg AAV8-Cre-eGFP, 2.3e11 vg AAV8-ROSA26-gRNAs, 1e11 vg AAV8-CMV-Cre-mCherry, 1e11 vg AAV8-LSP-Cre-mCherry, 3.16e11 vg AAV5-Cre-eGFP, and 3.16e11 vg AAV9-Cre-eGFP). Vehicle-injected mice received 100 µl of vector dilution buffer alone. The 100 µl volume was quickly injected into the lumen of the right jugular vein using a BD U-100 insulin syringe (Becton Dickinson). The skin overlying the exposed jugular vein was sutured and surgical recovery was monitored for 48 h post-injection. For perfusions and tissue collection, at 2 weeks post-injection mice were anesthetized with isoflurane and perfused with 15 ml of ice-cold phosphate-buffered saline (PBS) followed by 15 ml of ice-cold 4% paraformaldehyde (PFA) diluted in PBS. Brain, heart, lung, liver, kidney, spleen, skeletal muscle (quadriceps), and testes were collected from all mice. Cervical or thoracic lymph nodes, duodenum, and pancreas were also collected from all Ai14xSpCas9 mice. Organs were post-fixed in 4% PFA with a 24-h incubation at 4 °C and then cryoprotected with a 72-h incubation at 4 °C in a 30% sucrose solution (in PBS). After cryoprotection, organs were stored frozen at −80 °C.

### Histology

Cryoprotected mouse tissues were sectioned on a freezing microtome (brain, heart (left ventricle), lung, kidney, duodenum, and lymph node = 40 µm, liver and spleen = 10 µm, skeletal muscle (quadriceps), and pancreas = 40–60 µm, and testes = 50 µm). Tissue sections were rinsed with PBS, stained for nuclei with a 1:5000 dilution of Hoechst 33528 (in PBS), rinsed again in PBS, and then mounted with Fluoro-Gel (Electron Microscopy Sciences) and coverslipped. Mounted sections were imaged with a LEICA DM6000 B epifluorescence microscope using a ×10 or ×20 dry objective.

### Quantification of liver transduction

C57BL/6J and Ai14 mouse cryoprotected liver tissue was sectioned on a freezing microtome. Three non-consecutive (distances >500 um) sections were generated per mouse. Sections were rinsed with PBS, stained for nuclei with a 1:5000 dilution of Hoechst 33528 (in PBS), rinsed again in PBS, and mounted with Fluoro-Gel (Electron Microscopy Sciences), and coverslipped. Three non-overlapping images were obtained per section with a LEICA TCS SP8 X confocal microscope with an oil-immersion ×40 objective. Image acquisition was performed by an individual blinded to the treatment group. The total number of eGFP+ (C57BL/6J and Ai14), tdTomato+ (Ai14 only), and total hepatocytes (C57BL/6J and Ai14) was recorded for each image. The %eGFP+ and %tdTomato+ were calculated by dividing by the total number of hepatocytes per image.

### Measurement of vector copies per diploid genome equivalent

Genomic DNA from fixed and frozen C57BL/6J and Ai14 mouse tissues (liver, kidney, and spleen) was extracted using a DNeasy Blood & Tissue Kit (Qiagen) according to the manufacturer’s instructions. DNA concentration was measured via Qubit dsDNA HS Assay Kit (ThermoFisher Scientific) and DNA amount was calculated based on the concentration and volume of each sample. Absolute vector copies were measured in each sample using a standard curve and qPCR primer probe set designed to recognize the Cre transgene (Forward: 5′-TGA CGG TGG GAG AAT GTT AAT C-3′, Reverse: 5′-GCT ACA CCA GAG ACG GAA ATC-3′, Probe: /56-FAM/TCG CTC GAC/Zen/CAG TTT AGT TAC CCC/3IABkFQ/). Diploid genome equivalents (dge) were calculated for each sample based on DNA amount (5.43 pg of DNA per mouse dge)^28^.

### PCR editing assay

Forward: 5′-GCT GGT TAT TGT GCT GTC TCA TC-3′ and Reverse: 5′-CAT GAA CTC TTT GAT GAC CTC CTC-3′ primers were designed to bind upstream and downstream of the loxP-STOP-loxP region of the transgenic ROSA26 locus in the Ai14 mice. BIOTAQ (Bioline) PCR reactions were run using Ai14 mouse tissue (liver, kidney, or spleen) genomic DNA as a template. The PCR reaction cycle conditions were: 95 °C 5 min, [95 °C 30 s, 65 °C for 30 s, 72 °C for 45 s]×34, 72 °C 5 min, 4 °C hold. PCR products were separated and visualized on an agarose gel with an ethidium bromide stain. The full, uncropped gel images are provided in the Source Data file. The unedited version of this locus produces a 1142 bp PCR product. The edited version (Cre-recombined) of this locus produces a 271 bp PCR product. Both PCR products were purified, TOPO-cloned, and sequenced.

### Immunofluorescence

For podocyte (podoplanin) colocalization experiments: 30 µm Ai14 mouse kidney sections were blocked in 10% goat serum and 0.1% Triton X-100 in PBS for 1 h at room temperature. The sections were incubated with 1:250 (diluted in 2% goat serum and 0.1% Triton X-100 in PBS) primary syrian hamster anti-mouse podoplanin (Abcam, Cat No: 92319) for ~16 h at 4 °C. Sections were washed in PBS and then incubated with 1:2,000 secondary (diluted in 2% goat serum and 0.1% Triton X-100 in PBS) goat anti-syrian hamster IgG H+L (Alexa Fluor-488) (Abcam, Cat No: 180063) for 1 h at room temperature.

For endothelial cell (CD31) colocalization experiments: 30 µm Ai14 mouse kidney sections were blocked in 10% donkey serum and 0.1% Triton X-100 in PBS for 1 h at room temperature. The sections were incubated with 1:200 (diluted in 2% donkey serum and 0.1% Triton X-100 in PBS) of primary Rat anti-mouse CD31 (BD Biosciences, Cat. No: 557355) for ~16 h at 4 °C. Sections were washed in PBS and then incubated with 1:2,000 secondary (diluted in 2% donkey serum and 0.1% Triton X-100 in PBS) donkey anti-rat IgG H&L (Alexa Fluor-488) (ThermoFisher Scientific, Cat No: A-21208) for 1 h at room temperature. For mesangial cell (PDGFR-ß) colocalization experiments: 30 µm Ai14 mouse kidney sections were blocked in 10% goat serum and 0.1% Triton X-100 in PBS for 1 h at room temperature. The sections were incubated with 1:200 (diluted in 2% goat serum and 0.1% Triton X-100 in PBS) of primary rat anti-mouse CD140b (PDGFR-ß) (ThermoFisher Scientific, Cat. No: 14-1402-81) for ~16 h at 4 °C. Sections were washed in PBS and then incubated with 1:1000 secondary (diluted in 2% goat serum and 0.1% Triton X-100 in PBS) goat anti-rat IgG H&L (Alexa Fluor-488) (ThermoFisher Scientific, Cat No: A-11006) for 1 h at room temperature. Following secondary antibody incubation, all sections were washed in PBS, stained for nuclei with 1:5000 Hoechst 33528 (in PBS), and washed again in PBS. Sections were then mounted with Fluoro-Gel and coverslipped. Mounted sections were imaged on a LEICA TCS SP8 X confocal microscope with an oil-immersion ×63 objective.

### Splenocyte isolation and staining for flow cytometry

Ai14 mice were injected as described with vehicle or 1e11 vg of AAV8-Cre-eGFP. Mice were euthanized at 2 weeks post-injection and whole spleens were excised and processed using two complementary approaches that yielded similar populations of cells. In the first approach, whole spleens were digested mechanically by crushing between two frosted glass slides. In the second approach splenocytes were isolated from whole spleens using a modified version of a published stromal cell isolation protocol^29^. Briefly, the spleen was disrupted with forceps and then enzymatically digested with 1 mg/ml Collagenase IV (ThermoFisher Scientific) and 40 μg/ml DNAse I (Sigma Aldrich) for 30 min at 37 °C with shaking. The spleen fragments that remained were mechanically disrupted with pipetting and then further digested with 3.5 mg/ml Collagenase D (Sigma Aldrich) and 40 μg/ml DNase I (Sigma Aldrich) for 15 min at 37 °C with shaking. Spleen tissue fragments were then disaggregated to a single-cell suspension with additional pipetting. EDTA was added to promote maintenance of the single-cell suspension. This final cell suspension was passed through a 70 μm nylon mesh cell strainer. Cells from both protocols were depleted of red blood cells with ACK lysis buffer (ThermoFisher Scientific), and filtered through a 40 μm filter. The resulting single-cell suspension was then resuspended in PBS and stained with fixable Live/Dead Aqua (ThermoFisher) at 1:250 for 15 min at room temperature to exclude dead cells, followed by Fc receptor blockade with rat anti-mouse CD16/CD32 (clone 2.4G2; BD, cat #553142) at 1:100 in 0.1% PBS/BSA for 15 min at room temperature. Cells were then stained with antibodies all diluted at 1:100 in 0.1% PBS/BSA for 30 min at 4 °C specific for the following markers: CD45 (APC-Cy7 or BV785; clone 30-F11; Biolegend, cat #103116 or #103149), CD3 (AF700 or APC-Cy7; clone 17A2; Biolegend, cat #100216 or BD, cat #557596), CD19 (PerCP; clone 6D5; Biolegend, cat #115532), F4/80 (FITC; clone BM8; Biolegend, cat #123108), CD11c (BV605; clone N418; Biolegend, cat #117334), Ly6G (BUV395; clone 1A8; BD, cat #563978), CD31 (APC; clone MEC13.3; Biolegend, cat #102510). Flow cytometry data were acquired using both BD LSR Fortessa and Beckman Coulter Cytoflex LX analyzer instruments, and data were analyzed using FlowJo 10 software (FlowJo LLC).

### Reporting summary

Further information on research design is available in the Nature Research Reporting Summary linked to this article.

## Supplementary information

Supplementary Information

Reporting Summary

Source Data File

## Data Availability

The data that support the findings of this study are available from the corresponding author upon reasonable request. Source data underlying Figs. 2b-d, 3b-c, 4b-c, f-g are provided as a Source Data file.
