# Supplementary material for: Standard screening methods underreport AAV-mediated transduction and gene editing
Source: Nat Commun. 2019 Jul 30;10:3415. doi: 10.1038/s41467-019-11321-7 (PMC6667494; doi:10.1038/s41467-019-11321-7)
Supplement: Supplementary file 1 — Supplementary Information [file 41467_2019_11321_MOESM1_ESM.pdf]

Supplementary Information:

Standard screening methods underreport AAV-mediated transduction and gene editing

Lang et al.

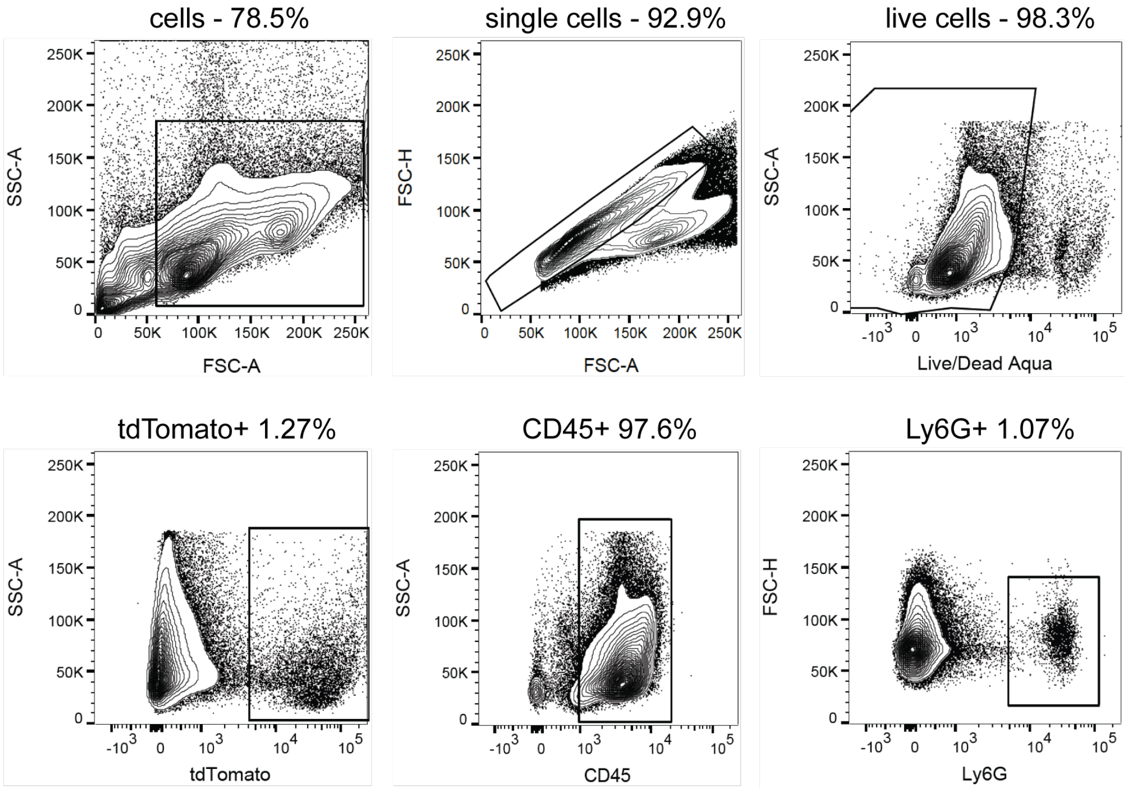

Supplemental Figure 1 | Flow cytometry gating strategy for Ai14 mouse splenocytes. Representative gating strategy for identification of tdTomato+/CD45+/Ly6G+ splenocytes isolated from an Ai14 mouse at two weeks post IV delivery of 1e11 vg AAV8-Cre-eGFP. This strategy underlies the flow data presented in Figure 4 of the main text. Specific marker fluorochromes are indicated in methods.

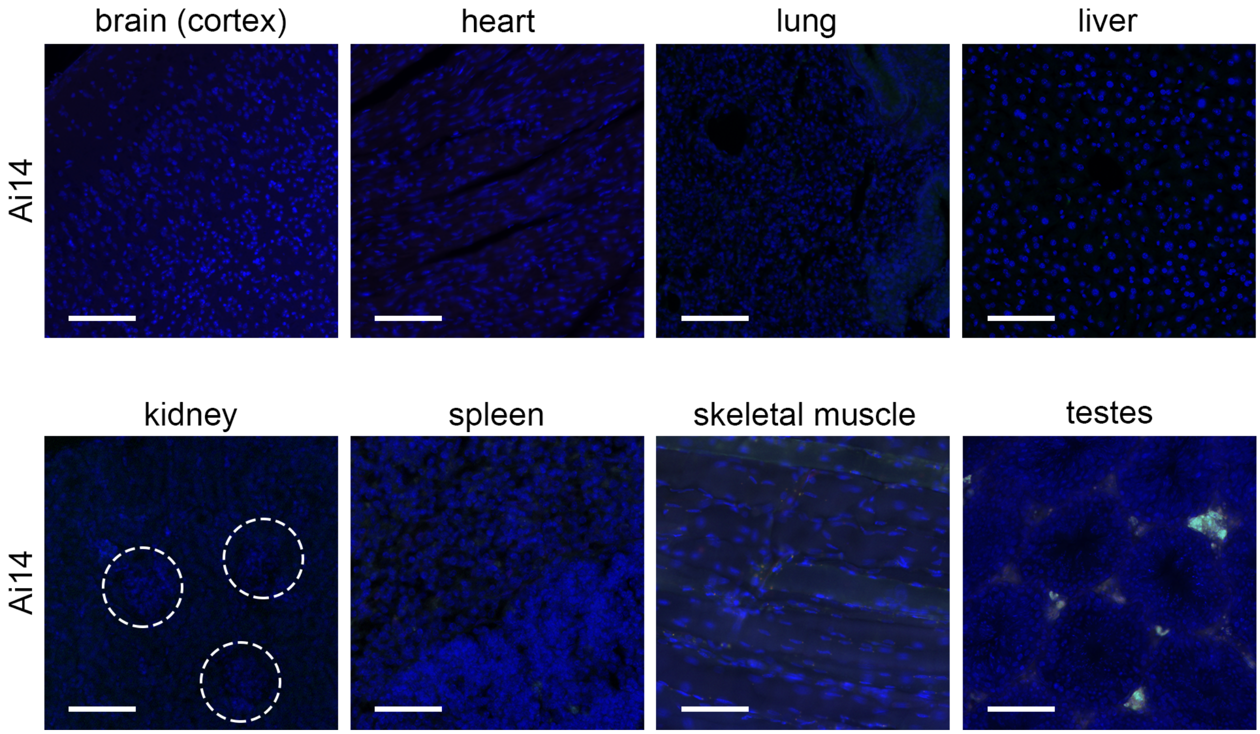

Supplemental Figure 2 | Ai14 mice do not express tdTomato following vehicle injection. Representative photomicrographs of tissues from Ai14 mice at 2 weeks post IV injection of vehicle. No eGFP expression is detected (green). No tdTomato expression is detected (red). Nuclei are shown (blue). Dashed circles highlight glomeruli. Autofluorescent material is present in testes. N=2 Ai14 mice. Scale bars = 100 μm.

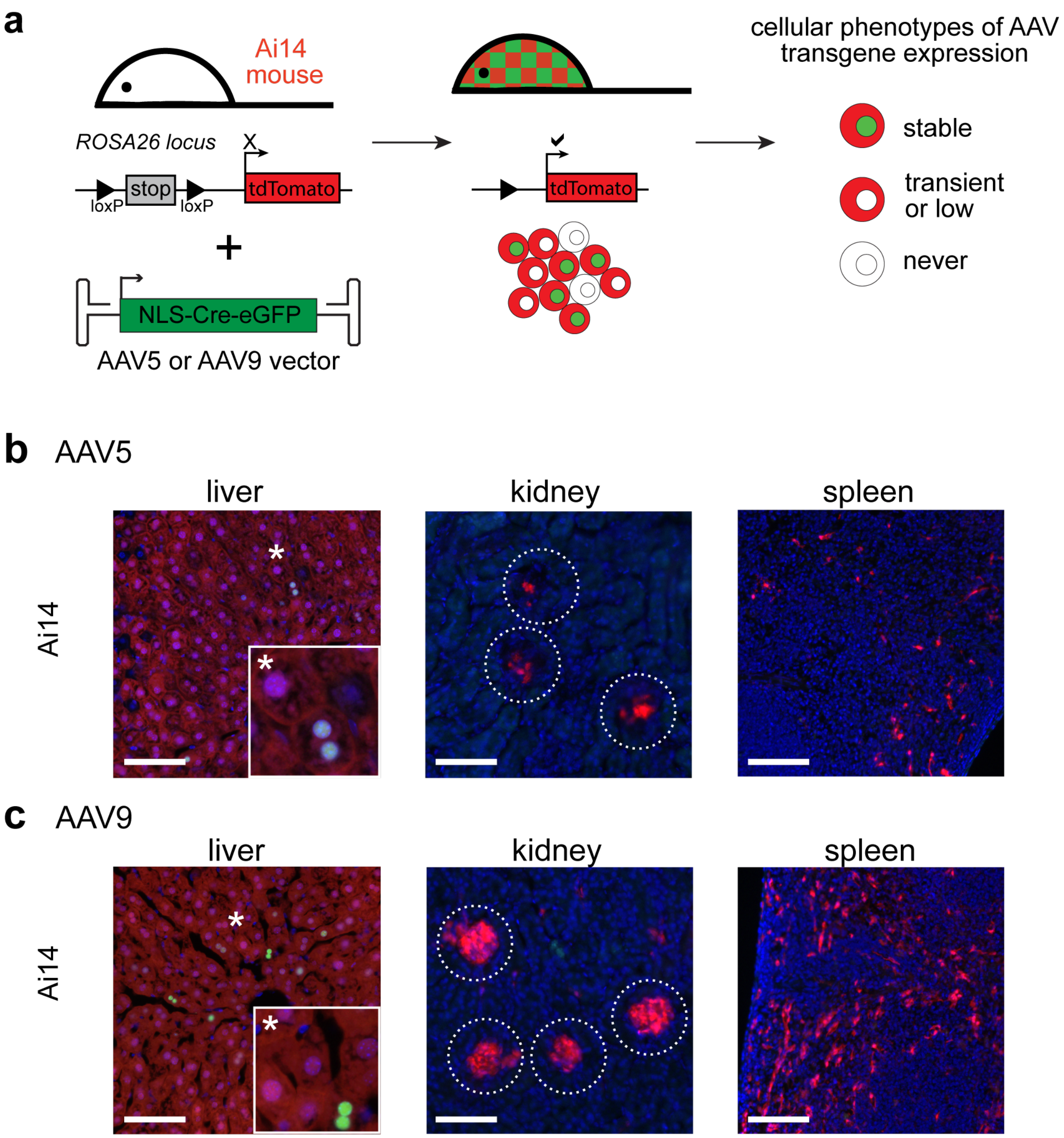

Supplemental Figure 3 | Ai14 mice capture AAV5 and AAV9 transgene expression in liver, kidney, and spleen. a, Injection of AAV5-Cre-eGFP or AAV9-Cre-eGFP into Ai14 reporter mice (containing an endogenous loxP-STOP-loxP-tdTomato locus) captures cells with stable, high transgene expression (green nuclei) as well as cells with transient or low expression of the AAV transgenes (Cre tagged tdTomato positive cells). **b**, Representative photomicrographs of Ai14 mouse tissue at two weeks post IV injection of 3.16e11 vg AAV2/5.CMV.HI.eGFP-Cre.WPRE.SV40 per mouse. **c**, Representative photomicrographs of Ai14 mouse tissue at two weeks post IV injection of 3.16e11 vg AAV2/9.CMV.HI.eGFP-Cre.WPRE.SV40 per mouse. eGFP (green) and tdTomato expression (red) and nuclei (blue) are shown. N=2 Ai14 mice for each vector. Dashed circles highlight glomeruli. Scale bars = 100 μm.

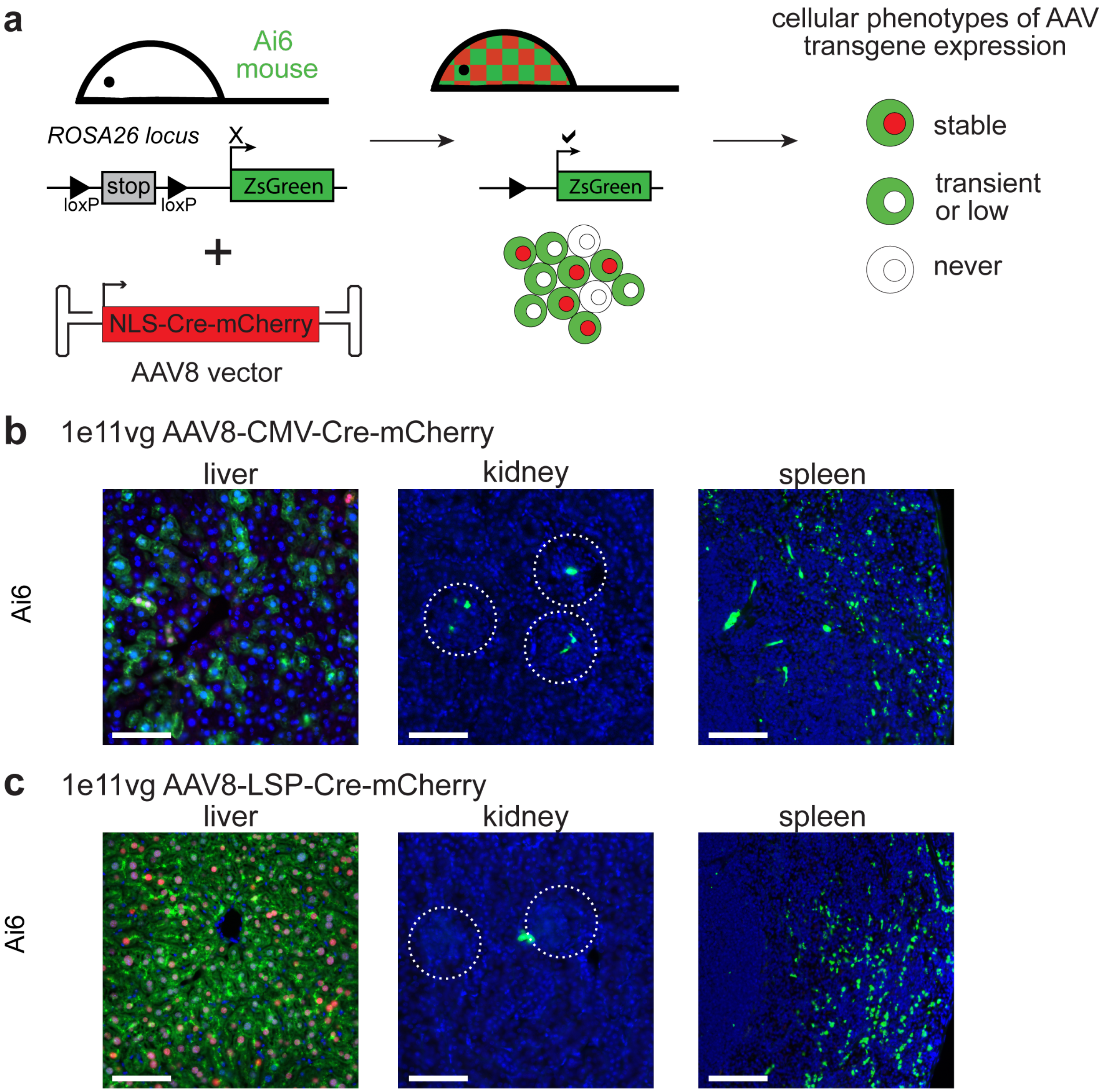

Supplemental Figure 4 | Ai6 reporter mice also capture AAV8 transgene expression with high sensitivity. a, Injection of AAV8-Cre-mCherry vector into Ai6reporter mice (containing an endogenous loxP-STOP-loxP-ZsGreen locus) captures cells with stable, high transgene expression (red nuclei) as well as cells with transient or low expression of the AAV transgenes (Cre tagged ZsGreen positive cells). **b**, Representative photomicrographs of Ai6 mouse tissue at two weeks post IV injection of 1e11 vg AAV8-CMV-Cre-mCherry per mouse. **c**, Representative photomicrographs of Ai6 mouse tissue at two weeks post IV injection of 1e11 vg AAV8-LSP-Cre-mCherry per mouse. AAV transgene expression is driven by a "liver-specific promoter." mCherry (red) and ZsGreen expression (green) and nuclei (blue) are detected. N=2 Ai6 mice for each vector. Scale bars = 100 µm.

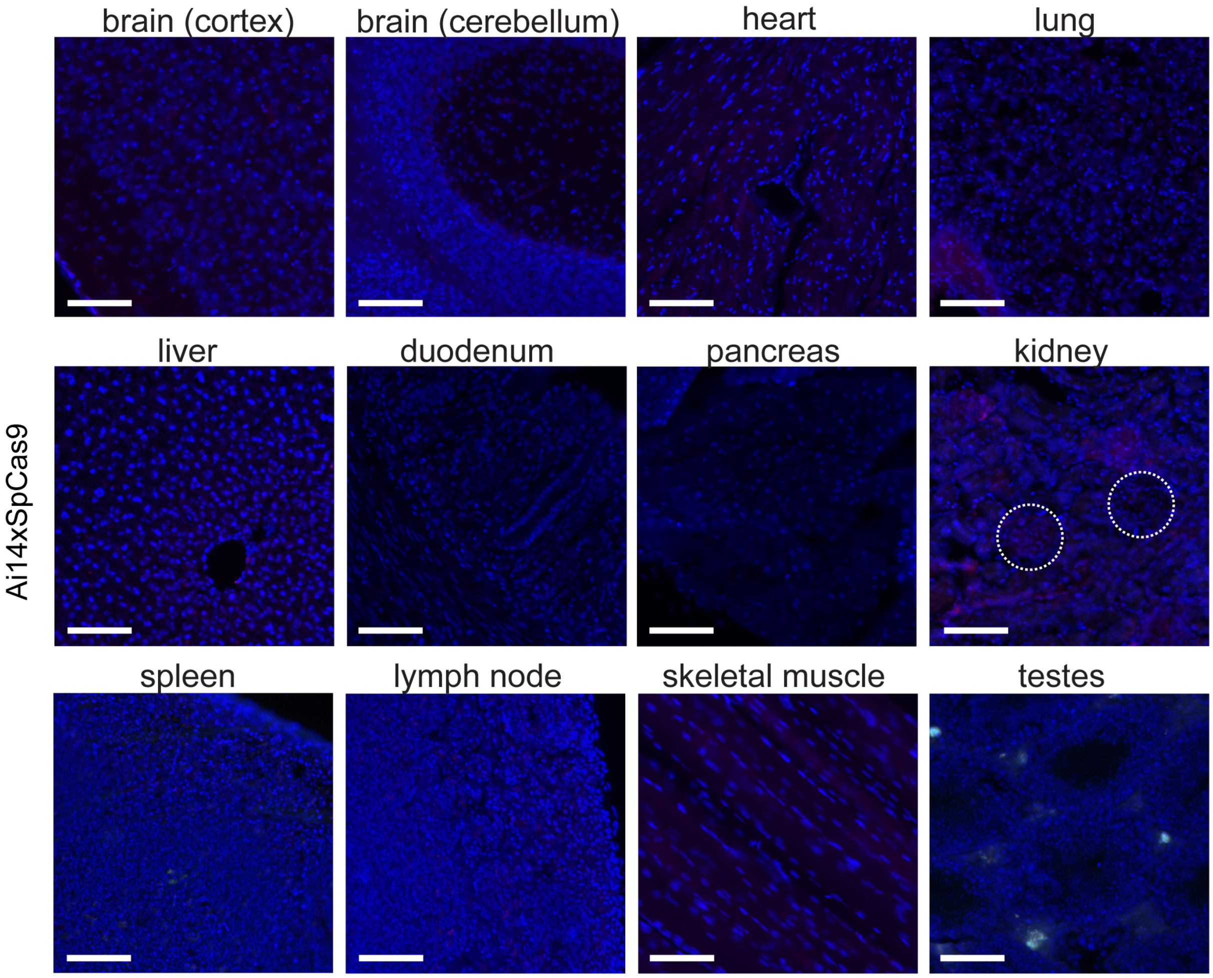

Supplemental Figure 5 | Non-injected Ai14xSpCas9 mice do not express tdTomato at baseline. Representative photomicrographs of tissues from non-injected male, Ai14xSpCas9 mice. No tdTomato expression is detected (red). Nuclei are shown (blue). Dashed circles highlight glomeruli. Autofluorescent material is present in testes. N=2 Ai14xSpCas9 mice. Scale bars = 100 µm.
