# Supplementary material for: Standard screening methods underreport AAV-mediated transduction and gene editing
Source: Nat Commun. 2019 Jul 30;10:3415. doi: 10.1038/s41467-019-11321-7 (PMC6667494; doi:10.1038/s41467-019-11321-7)
Supplement: Supplementary file 2 — Reporting Summary [file 41467_2019_11321_MOESM2_ESM.pdf]

nature research

Corresponding author(s): Beverly Davidson

Last updated by author(s): Jun 15, 2019

Reporting Summary

Nature Research wishes to improve the reproducibility of the work that we publish. This form provides structure for consistency and transparency in reporting. For further information on Nature Research policies, see Authors & Referees and the Editorial Policy Checklist.

Statistics

For all statistical analyses, confirm that the following items are present in the figure legend, table legend, main text, or Methods section.

n/a	Confirmed	
☐	☒	The exact sample size (*n*) for each experimental group/condition, given as a discrete number and unit of measurement
☐	☒	A statement on whether measurements were taken from distinct samples or whether the same sample was measured repeatedly
☐	☒	The statistical test(s) used AND whether they are one- or two-sided<br>*Only common tests should be described solely by name; describe more complex techniques in the Methods section.*
☒	☐	A description of all covariates tested
☒	☐	A description of any assumptions or corrections, such as tests of normality and adjustment for multiple comparisons
☒	☐	A full description of the statistical parameters including central tendency (e.g. means) or other basic estimates (e.g. regression coefficient) AND variation (e.g. standard deviation) or associated estimates of uncertainty (e.g. confidence intervals)
☒	☐	For null hypothesis testing, the test statistic (e.g. *F*, *t*, *r*) with confidence intervals, effect sizes, degrees of freedom and *P* value noted<br>*Give P values as exact values whenever suitable.*
☒	☐	For Bayesian analysis, information on the choice of priors and Markov chain Monte Carlo settings
☒	☐	For hierarchical and complex designs, identification of the appropriate level for tests and full reporting of outcomes
☒	☐	Estimates of effect sizes (e.g. Cohen's *d*, Pearson's *r*), indicating how they were calculated

Our web collection on statistics for biologists contains articles on many of the points above.

Software and code

Policy information about availability of computer code

Data collection	Microscopy: Leica Application Suite X (Leica Microsystems CMS GmbH)<br>qPCR: Bio-Rad CFX Manager (Bio-Rad Laboratories)<br>Agarose gel imaging: Image Lab (Bio-Rad Laboratories)<br>Flow cytometry: FACSDiva (BD Biosciences) and CytExpert (Beckman Coulter Life Sciences)
Data analysis	Microscopy: Leica Application Suite X (Leica Microsystems CMS GmbH) and ImageJ (Fiji distribution)<br>qPCR: Microsoft Excel and GraphPad PRISM (GraphPad Software, Inc.)<br>Agarose gel imaging: Image Lab (Bio-Rad Laboratories)<br>Flow cytometry: FlowJo 10 software (FlowJo LLC)

For manuscripts utilizing custom algorithms or software that are central to the research but not yet described in published literature, software must be made available to editors/reviewers. We strongly encourage code deposition in a community repository (e.g. GitHub). See the Nature Research guidelines for submitting code & software for further information.

Data

Policy information about availability of data

All manuscripts must include a data availability statement. This statement should provide the following information, where applicable:

- Accession codes, unique identifiers, or web links for publicly available datasets
- A list of figures that have associated raw data
- A description of any restrictions on data availability

The data that support the findings of this study are available from the corresponding author upon reasonable request.

Field-specific reporting

Please select the one below that is the best fit for your research. If you are not sure, read the appropriate sections before making your selection.

☒ Life sciences ☐ Behavioural & social sciences ☐ Ecological, evolutionary & environmental sciences

For a reference copy of the document with all sections, see nature.com/documents/nr-reporting-summary-flat.pdf

Life sciences study design

All studies must disclose on these points even when the disclosure is negative.

Sample size	No sample size calculations were performed for this study . The exact number of mice used for each experiment is indicated in the figure legends.
Data exclusions	No data were excluded from this study.
Replication	Our results were consistent across several independent mice for each vector and/or vector dose group.
Randomization	The study was limited to adult (>9 weeks), male mice from each strain to help control for variability due to age and biological sex, but additional randomization was not performed.
Blinding	The authors were blinded to mouse sample ID and dose group during quantification of transduction in mouse liver sections.

Reporting for specific materials, systems and methods

We require information from authors about some types of materials, experimental systems and methods used in many studies. Here, indicate whether each material, system or method listed is relevant to your study. If you are not sure if a list item applies to your research, read the appropriate section before selecting a response.

Materials & experimental systems

n/a	Involved in the study	
☐	☒	Antibodies
☒	☐	Eukaryotic cell lines
☒	☐	Palaeontology
☐	☒	Animals and other organisms
☒	☐	Human research participants
☒	☐	Clinical data

Methods

n/a	Involved in the study	
☒	☐	ChIP-seq
☐	☒	Flow cytometry
☒	☐	MRI-based neuroimaging

Antibodies

Antibodies used	Immunofluorescence experiments:<br>For podocytes: primary syrian hamster anti-mouse podoplanin (Abcam, Cat No: 92319) and secondary goat anti-syrian hamster IgG H+L (Alexa Fluor-488) (Abcam, Cat No: 180063).<br>For endothelial cells: primary rat anti-mouse CD31 (BD Biosciences, Cat. No: 557355) and secondary donkey anti-rat IgG H&L (Alexa Fluor-488) (ThermoFisher Scientific, Cat No: A-21208).<br>For mesangial cells: primary rat anti-mouse CD140b (PDGFR-ß) (ThermoFisher Scientific, Cat. No: 14-1402-81) and secondary goat anti-rat IgG H&L (Alexa Fluor-488) (ThermoFisher Scientific, Cat No: A-11006).<br><br>Flow cytometry experiments:<br>Fc receptor blockade: rat-anti mouse CD16/CD32 (BD, clone 2.4G2).<br>Cell markers: CD45 (clone 30-F11; Biolegend), CD3 (clone 17A2; Biolegend), CD19 (clone 6D5; Biolegend), F4/80 (clone BM8; Biolegend), CD11c (clone N418; Biolegend), Ly6G (clone 1A8; BD), CD31 (clone 390; Biolegend).
Validation	Validation of all antibodies is described by the manufacturers. For immunofluorescence experiments, vehicle-injected mouse tissues and secondary antibody only conditions were used as negative controls.

Animals and other organisms

Policy information about studies involving animals; ARRIVE guidelines recommended for reporting animal research

Laboratory animals	The following mouse strains were obtained from Jackson Laboratories:<br>C57BL/6J (C57BL/6J, Stock No: #000664)<br>Ai14 (B6.Cg-Gt(ROSA)26Sortm14(CAG-tdTomato)Hze/J, Stock No: #007914)

	Ai6 (B6.Cg-Gt(ROSA)26Sortm6(CAG-ZsGreen1)Hze/J, Stock No: #007906)<br>SpCas9 (B6J.129(Cg)-Igs2tm1.1(CAG-cas9*)Mmw/J, Stock No: #028239)<br>The following mouse strain was generated in the lab:<br>Ai14 and SpCas9 mice were crossed to generate Ai14xSpCas9 offspring.
Wild animals	This study did not involve wild animals.
Field-collected samples	This study did not involve samples collected from the field.
Ethics oversight	All animal procedures were approved by The Children's Hospital of Philadelphia Institutional Animal Care and Use Committee.

Note that full information on the approval of the study protocol must also be provided in the manuscript.

Flow Cytometry

Plots

Confirm that:

☐ The axis labels state the marker and fluorochrome used (e.g. CD4-FITC).

☒ The axis scales are clearly visible. Include numbers along axes only for bottom left plot of group (a 'group' is an analysis of identical markers).

☒ All plots are contour plots with outliers or pseudocolor plots.

☒ A numerical value for number of cells or percentage (with statistics) is provided.

Methodology

Sample preparation	Ai14 mice were euthanized at two weeks post-injection and whole spleens were excised and processed using two complementary approaches (described in manuscript) that yielded similar populations of cells.
Instrument	Data were obtained using BD LSR Fortessa and Beckman Coulter Cytoflex LX analyzer instruments.
Software	Data were collected with FACSDiva (BD Biosciences) or CytExpert (Beckman Coulter Life Sciences) software and then analyzed using FlowJo 10 software (FlowJo LLC).
Cell population abundance	For each AAV-injected mouse, at least 1 million live cell events and at least 15,000 tdTomato+ live cell events were analyzed for the multistained condition. At least 30,000 live cell events were analyzed for the unstained and fluorescence minus one conditions.<br>For each vehicle-injected mouse, at least 30,000 live cell events were analyzed for multistained, unstained, and fluorescence minus one conditions.
Gating strategy	FSC-A and SSC-A were used to exclude debris. FSC-H and FSC-A were used to exclude doublets. Live/Dead Aqua was used to exclude dead cells. Fluorescence minus one controls were used to establish positive thresholds for each cell marker. Vehicle-injected Ai14 mice were used as negative controls for tdTomato expression.

☒ Tick this box to confirm that a figure exemplifying the gating strategy is provided in the Supplementary Information.
